# Pressure Perturbation Studies of Noncanonical Viral Nucleic Acid Structures

**DOI:** 10.3390/biology10111173

**Published:** 2021-11-12

**Authors:** Judit Somkuti, Orsolya Réka Molnár, Anna Grád, László Smeller

**Affiliations:** Department of Biophysics and Radiation Biology, Semmelweis University, 1094 Budapest, Hungary; somkuti.judit@med.semmelweis-univ.hu (J.S.); molnar.orsolya@med.semmelweis-univ.hu (O.R.M.); grad.anna@med.semmelweis-univ.hu (A.G.)

**Keywords:** G-quadruplex, hepatitis B, DNA, oligo, FRET, FTIR, spectroscopy, pressure, volume change, TMPyP4

## Abstract

**Simple Summary:**

It is well known that nucleic acids adopt a double-helical structure that is essential in the storage and replication of genetic code. However, noncanonical structures of DNA are less explored. The most important of these structures are the G-quadruplexes, which are formed by guanine-rich sequences of the genome. These G-quadruplexes were found in several crucial positions of the genome; they regulate important processes such as cell proliferation and death. Here we are investigating the quadruplex structures appearing in the genome of the hepatitis B, whose infection is among the ten leading causes of death. Our unique approach—the high-pressure technique—allowed us to characterize the stability of these quadruplexes in wide temperature and pressure ranges. Pressure experiments gave us the volume changes occurring at the unfolding, e.g., the embedded volume of the folded structure. Volumetric parameters are especially important because the space available for molecules is quite limited in the crowded environment of the cell. We also investigated how the stability of the viral quadruplexes can be increased by the binding of TMPyP4, a special ligand developed for cancer therapy.

**Abstract:**

G-quadruplexes are noncanonical structures formed by guanine-rich sequences of the genome. They are found in crucial loci of the human genome, they take part in the regulation of important processes like cell proliferation and cell death. Much less is known about the subjects of this work, the viral G-quadruplexes. We have chosen three potentially G-quadruplex-forming sequences of hepatitis B. We measured the stability and the thermodynamic parameters of these quadruplexes. We also investigated the potential stabilization of these G-quadruplexes by binding a special ligand that was originally developed for cancer therapy. Fluorescence and infrared spectroscopic measurements were performed over wide temperature and pressure ranges. Our experiments indicate the small unfolding volume change of all three oligos. We found a difference between the unfolding of the 2-quartet and the 3-quartet G-quadruplexes. All three G-quadruplexes were stabilized by TMPyP4, which is a cationic porphyrin developed for stabilizing the human telomere.

## 1. Introduction

**Figure 1:**
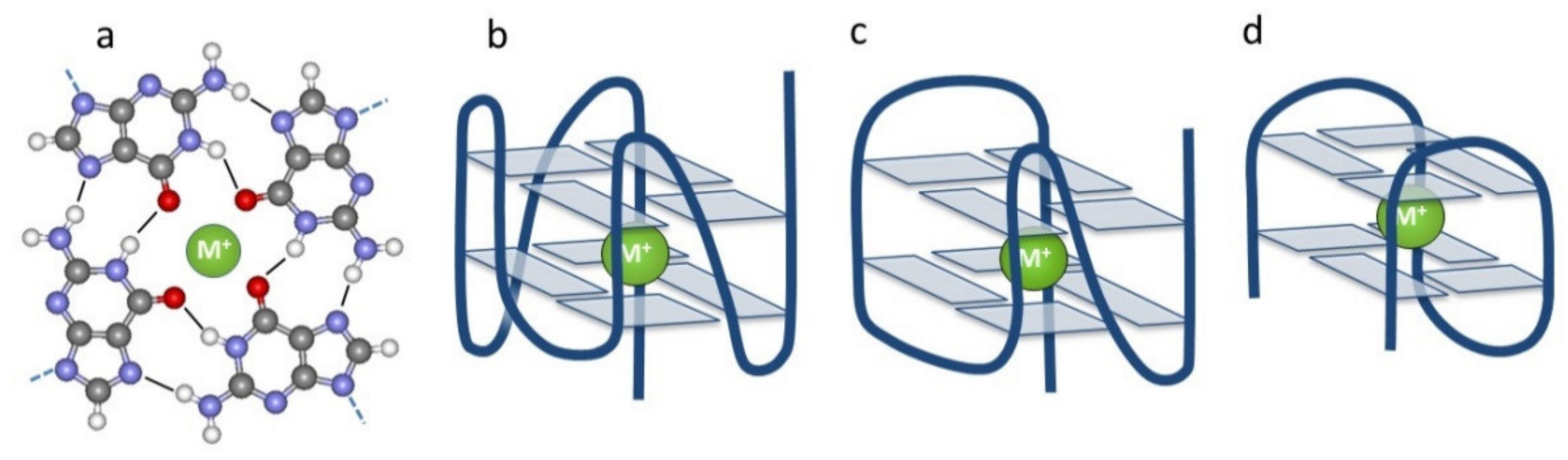
(**a**) Structure of a G-quartet formed by four guanine bases. The figure was made by the Viewerlite 4.2 program, selecting one quartet from the 2HY9 pdb structure. (**b**–**d**) Typical structures of a two-quartet GQ. The panels show the parallel, hybrid and basket forms respectively.

Guanine-rich nucleic acid sequences can form non-canonical structures. One of the most important among these structures are the four-stranded G-quadruplex (GQs), in which four guanines are arranged in one plane. They are connected by Hoogsteen-type hydrogen bonds instead of the Watson–Crick-type ones (Figure 1), and they are stabilized by eight hydrogen bonds. A GQ is typically composed of two or three G-quartets. A central metal ion is essential, as it is necessary to stabilize the structure. GQs can have a number of different conformations they can be formed by one, two or four different strands. The monomers are the most important forms, in these structures one nucleic acid chain is folded to form the GQ. As for the orientation of the strands, they can appear in parallel, antiparallel or hybrid forms [1,2]. GQs, in vitro and in vivo, can interact with various ions and molecules that give them special biological significance. In vivo, GQs take part in the regulation of gene expression; in vitro, they are used in analytical biochemistry [3]. Their in-vivo relevance is mostly related to the stabilization of the DNA or RNA. GQs came into the focus of the research, when their abundance in the telomere region of the human genome was reported [4,5]. Formation of GQs in this region inhibits the telomerase activity. Additionally, they were found in the promoter region of several oncogenes [6,7], which made them a potential target for cancer treatment [4,8]. Recently, potentially GQ-forming sequences were found in several viral genomes, and it opens the possibility to target viral GQs using molecules developed for cancer research. Additionally, an interaction between viral proteins and GQs was also reported recently, which underlines the importance of GQs in viral research [9].

In this paper we report the evaluation of three GQ-forming sequence of the hepatitis B virus (HBV). HBV is one of the most frequent chronic viral infections, around 350–400 million people are currently infected worldwide [10]. HBV infection was among the ten leading causes of death in 2010 [11]. HBV has a small circular DNA genome with a length of approximately 3.2 kilobase pairs [10,11,12,13]. HBV has four overlapping reading frames that encode seven proteins (including S for surface or envelope gene, C for the core gene and P for the polymerase gene) [12,14].

Lavezzo et al. [15] were searching for potential GQ-forming sequences in genomes of several viruses. For the HBV, the highest G-score was associated to the GGC TGG GGC TTG GTC ATG GGC CAT CAG (NC_003977.2:1204..1230 (+strand)) sequence, which can be found in the coding region of the polymerase protein.

Several other potential GQ-forming sequences with slightly lower score were also identified. Biswas et al. [12] found a conserved sequence containing GGG tetrads in the promoter region of the envelope-coding gene, the S protein of the virus. This GGG AGT GGG AGC ATT CGG GCC AGG G sequence is also worthy of investigation. Another promising GQ-prone sequence might be the GGG TGG CTT TGG GGC ATG G (NC_003977.2 NC_003977.2:1886..1904 (+strand)) which can be found in the C protein’s signaling region in the virus.

In experimental practice, pressure is a highly overlooked variable of thermodynamics, although it is equally important as temperature [16,17,18,19,20,21,22]. The low number of high-pressure studies can be partially explained by technical difficulties. Experiments performed at few kilobars are considered by some as non-physiological. Of course, high temperatures or a few moles of GuHCl—which are frequently used in stability studies of macromolecules—are not physiological either. However, high-pressure experiments give the unique possibility of revealing the volumetric properties of the system under study.

The usefulness of high-pressure experiments has been proven in a wide range of publications [22,23,24,25,26,27,28,29,30]. According to the Le Chatelier’s principle, the pressurized system prefers the state that has the smallest volume. This is how protein unfolding is induced by pressure [31,32,33]. Typically 5 kbar pressure is enough to unfold an average protein, like myoglobin [34], although there are much more stable ones as well [35]. Intermolecular interactions are even more sensitive to pressure; typically 2 kbar leads to disaggregation of oligomeric structures [29,34,36]. Lipid-phase transition lines are also shifting with pressure, which is again a nice example for the Le Chatelier’s principle: pressure stabilizes the more tightly packed gel phase [37,38].

Nucleic acids were treated as pressure insensitive for a long time. The unwinding of the double helix structures is hardly influenced by pressure, but noncanonical structures, like GQs and i-motifs show volume changes during their unfolding [22,26,39,40,41]. Therefore, they are appropriate for such high-pressure studies that can provide volumetric data. The volume change of the folded GQ structure allows characterization of the tightness and stability of this noncanonical form.

This paper focuses on the stability and volumetric parameters of the above mentioned three GQ-prone sequences of the hepatitis B virus. The investigation of these structures is of vital importance for designing methods wherein the life cycle of the virus is influenced by targeting these noncanonical structure-forming parts of the genome.

## 2. Materials and Methods

Three oligonucleotides—named HepB1, HepB2 and HepB3—were investigated. Their sequences are: HepB1: GGC TGG GGC TTG GTC ATG GGC CAT CAG, HepB2: GGG AGT GGG AGC ATT CGG GCC AGG G HepB3: TTG GGT GGC TTT GGG GCA TGG AC. The oligonucleotides were purchased from IDT (Coralville, IA, USA) and Sigma-Aldrich Kft (Budapest, Hungary).

For the fluorescence studies, the oligos were labeled with the FRET pair FAM and TAMRA. (FRET: Förster resonance energy transfer, FAM: Carboxyfluorescein CAS Number: 3301-79-9, TAMRA^TM^: Tetramethylrhodamine) The labeling was performed by IDT or Sigma-Aldrich, where the oligos were synthetized. The oligos were obtained from the manufacturers in lyophilized form.

For the fluorescence experiments, the HepB_FRET samples were first dissolved in MilliQ water in a concentration of 100 μM, according to the suggestion of IDT. This stock solution was kept frozen and diluted with an appropriate buffer during sample preparation. The final concentration of the oligos in the samples was 1 μM, unless stated otherwise. For the heating experiments, 100 mM K-phosphate buffer (pH 7.4) was used, because of its insensitivity to temperature changes. For experiments with Li^+^ and Rb^+^, Tris buffer was used. TMPyP4 (meso-5,10,15,20-Tetrakis-(N-methyl-4-pyridyl)porphine) was purchased from Merck KGaA, (Darmstadt, Germany) and ChemCruz (Dallas, TX, USA).

In the infrared experiments much higher concentration of HepB was required. The oligos were dissolved in D_2_O-based Bis-Tris buffer (100 mM, pD 7.4) in a concentration of 20 mg/mL. The metal ion concentrations were 100 mM. All chemicals not specified above differently were purchased from Sigma-Aldrich.

Fluorescence experiments were performed as described earlier in details [25,42]. Fluorolog-FL3 fluorimeter (Horiba Jobin Yvon, Longjumeau, France) was used mainly for the temperature scans. It was equipped with a programmable temperature-controlled cell (DI instruments, Budapest, Hungary). An HH802U thermometer and the corresponding software from Omega were used to record the temperature by a thermocouple directly in the cuvette (Omega Engineering Inc., Norwalk, CT, USA). The temperature was increased by 0.2 °C/min. Spectra were recorded every 5 min, resulting in spectra of ca. 1 °C intervals. Fluorescence intensity was divided with the signal of the reference diode of the spectrometer in order to standardize the experiments. For the high-pressure experiments, a homemade reflection-mode diamond cell was adopted for the sample holder. The pressure was measured by recording the ruby fluorescence. A small ruby chip was placed into the pressure cell, and it was excited by a green HeNe laser (Coherent, Santa Clara, CA, USA). The emitted light was detected by a CCD camera (Andor, Belfast, UK) attached to a THR1000 monochromator (Jobin Yvon, Longjumeau, France).

FTIR spectra were measured with a Bruker Vertex 80v spectrometer. A total of 256 spectra were averaged at a 2 cm^−1^ resolution. D_2_O buffer was used as solvent, in order to avoid the large absorption band of water around 1640 cm^−1^. The samples were measured in a temperature-controlled diamond anvil cell (Diacell, Leicester, UK). The pressure was measured using the 983 cm^−1^ line of BaSO_4_ [43]. The experiments were performed by increasing the temperature with a rate of 12 °C/hour at constant pressure. Spectra were recorded in 5 min intervals, i.e., in ca. 1 °C steps.

CD (circular dichroism) spectra were recorded with Jasco J-720 spectrometer in a 1 mm cuvette with 0.1 nm steps. The spectra were smoothed with Fourier filter.

The transition temperature was determined by fitting the temperature dependence of spectral parameters by the following sigmoidal function:

(1) $$y(T)=a+bT+\frac{\Delta a+\Delta bT}{1+\exp\left(\frac{\Delta H}{R}\left(\frac{1}{T}-\frac{1}{T_{m}}\right)\right)}$$

Here, *y* is the physical parameter to be fitted (e.g., fluorescence intensity, ratio of fluorescence intensities at two different wavelengths or absorbance at certain wavenumber of the infrared spectrum), *a* and *b* are the parameters describing the linear dependence of *y*(*T*) below the transition, *T* is the thermodynamic temperature, ∆*a* and ∆*b* are the changes of *a* and *b* during the transition, ∆*H* is the enthalpy change, *R* is the universal gas constant, and *T*_m_ is the transition midpoint.

The Clausius–Clapeyron equation was used to calculate the volume changes taking place during the pressure experiments:

(2) $$\Delta V=\frac{\Delta H}{T_{m}}\frac{dT_{m}}{dp}$$

It has to be mentioned, that in this paper the volume change is defined as the unfolding volume, that means Δ*V* = *V*_unfolded_ − *V*_folded_.

## 3. Results and Discussion

### 3.1. Temperature Stability of HepB1-3 Detected by FRET

**Figure 2:**
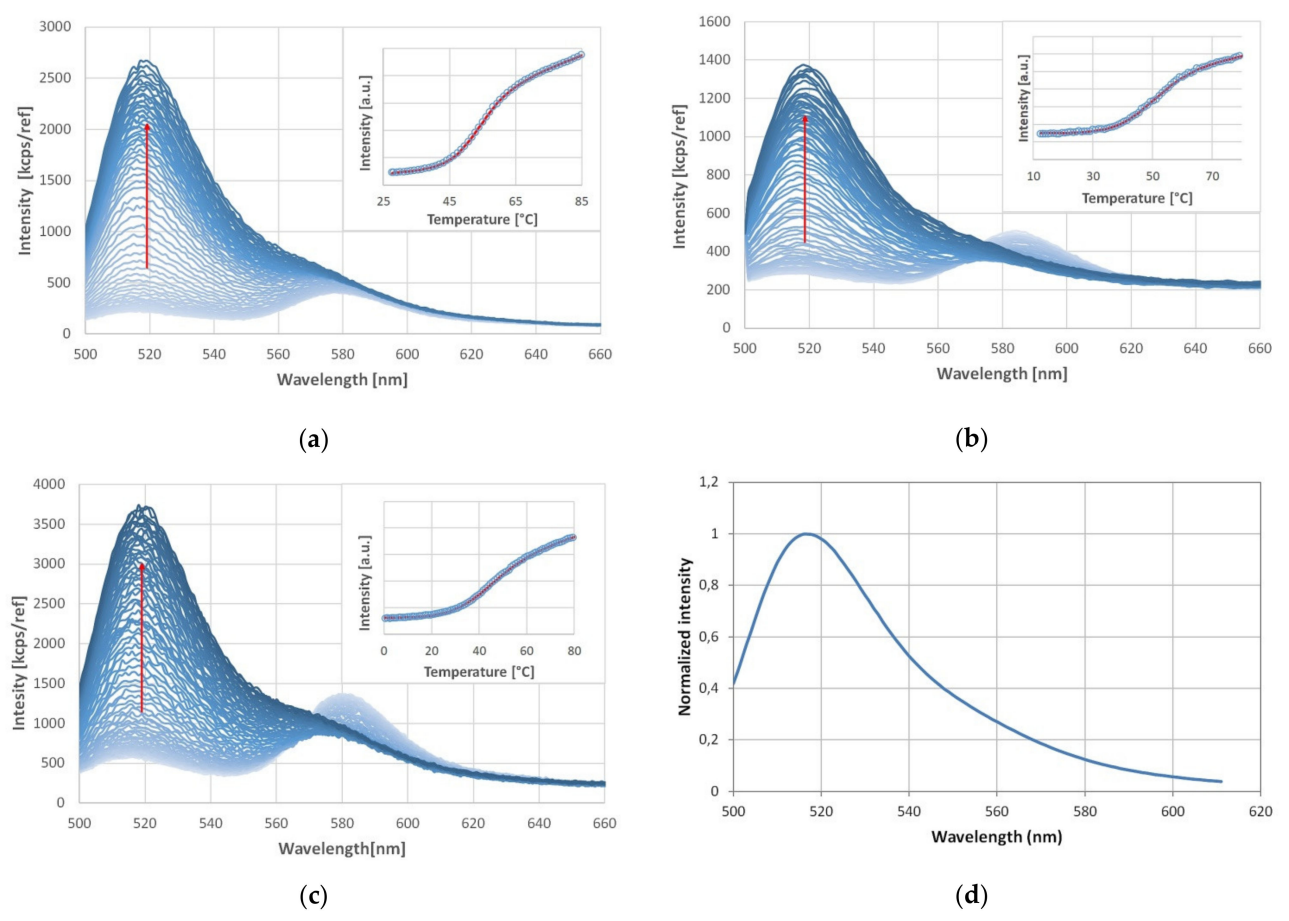
Fluorescence spectra of (**a**) HepB1, (**b**) HepB2, (**c**) HepB3 and (**d**) human telomere (Htel) at atmospheric pressure. The HepB oligos are labeled by the FRET pair FAM (as donor) and TAMRA (as acceptor), Htel was labeled only with FAM. The fluorescence intensity is divided with the signal of the reference diode of the spectrometer in order to standardize the experiments. The lowest temperature spectrum has the lightest color while the high temperature one is dark. Additionally a red arrow shows the direction of the spectral changes during temperature increase. The inserts in (**a**–**c**) show the fit of the donor intensity with the function of Equation (1). The oligos were measured in presence of 140 mM K-phosphate buffer (pH 7.4). The oligo labeled with FAM only can be seen in panel (**d**) to show the case, when there is no energy transfer.

Figure 2a–c shows the change of the fluorescence spectra of the three oligos during the temperature scan. All the oligos were labeled by FRET pair, and K^+^ ions were present to stabilize the GQs. An increase of the donor fluorescence intensity at 520 nm can be observed while the fluorescence of the acceptor is decreasing. The intensity at the acceptor position seems to be constant in case of HepB1, but this is due to the emission of the donor at 580 nm. Indeed, the high temperature spectra contain purely the donor emission, and the acceptor emission is completely lost. For comparison, a spectrum of GQ with FAM labeling only is shown in Figure 2d. In this case energy transfer is not possible, so it shows the spectrum of the donor without energy transfer. This spectrum is quite similar to the high temperature spectra on Figure 2a–c. Consequently, we can state the absence of—or at least a considerable reduced—energy transfer in the high-temperature spectra of HepB1-3. The increase of the donor fluorescence and the simultaneous decrease of the acceptor fluorescence clearly indicates the unfolding of the GQ structure at high temperatures. The Förster distance of the FAM-TAMRA FRET pair is 5.5 nm. In case of the folded GQ, the distance between the quartets is around 0.3 nm, while the width of the structure is around 2.5 nm. The distance between the first and last phosphorus atoms is around 2.5 nm in a three-quartet hybrid GQ. Since a 3D structure is not available for any HepB variants, these values were calculated using the 2HY9.pdb structure, which is a hybrid-form GQ found in human telomere sequences [44]. In this form the two terminal bases are at the different sides of the three-dimensional structure, having the biggest possible distance from each other. This means, that the highest possible distance in the two terminal bases in the folded GQ is much smaller than the Förster distance of the FRET pair we used. Therefore, the energy transfer in the low temperature spectra shows unequivocally a folded GQ form. Our HepB1, HepB2 and HepB3 oligos had 27, 25 and 23 bases respectively. The contour lengths of oligos containing 27, 25 and 23 bases were 8.1, 7.5 and 6.9 nm, which, together with the lengths of the linker of the chromophores (ca. 2 and 1 nm), were definitely larger than the Förster distance of the FAM-TAMRA pair. This means the loss of the energy transfer, i.e., the high intensity of the donor fluorophore is a clear sign of the unfolding of the GQ structure.

The folded state can be characterized by the relative FRET efficiency (*E*_rel_) [45]. It is defined as:

*E*_rel_ = *I*_a_/(*I*_a_ + *I*_d_) (3)

where *I*_a_ and *I*_d_ are the fluorescence intensities at the acceptor and donor positions. Since the donor also emits slightly at the acceptor position, the value 0.11 means complete absence of the FRET.

The *E*_rel_ values are summarized in Table 1. All the folded values are around 0.65. The small variation of *E*_rel_ suggests that the folded forms have the same or at least similar folded conformation. In our earlier circular dichroism spectroscopic studies, we found a hybrid structure for the potassium-stabilized HepB1 [46]. We measured the CD spectra of HepB2 and HepB3 in presence of K^+^ ion. These spectra are shown in the Supplementary Materials (Figure S1a,b). Based on these spectra we can rationalize the hybrid structure for all the HepB variants in the presence of K^+^ ion [47,48]. The small *E*_rel_ values at high temperatures indicate the single-stranded (unfolded) conformation. Oliva et al. [45] measured *E*_rel_ = 0.33 for the unfolded Htel, which was shorter than our oligos. Their *E*_rel_ value for the folded hybrid form was a bit higher than our values, but HepB1 was proven to form a hybrid structure in presence of potassium ion. The discrepancy can be explained, again, by the different oligo lengths.

We evaluated the donor emission intensity to characterize the unfolding quantitatively. The donor intensity versus temperature is plotted in the inserts. A sigmoid curve (Equation (1)) was fitted, which was obtained from the two state thermodynamics. These are shown in the inserts. The transition temperatures were 53.8, 51.9 and 41.4 °C for HepB1, HepB2 and HepB3 respectively. It is noteworthy that HepB1 is the most stable, although HepB2 would be able to form a three-quartet GQ, which is considered more stable than the two-quartet one. Transition temperatures around 50 °C are typical for two-quartet GQs. As for the unfolding temperature of the two-quartet thrombin-binding aptamer (TBA), 53 °C was measured [41], while the three-quartet Htel unfolds at around 65 °C [42,49]. This means that either HepB2 does not form a three-quartet GQ, or it was somehow destabilized. Guedin et al. [50] investigated the effect of the loop length, and they found a clear destabilization caused by long loops. Since HepB2 has a long loop in the middle, this can be the reason for its low stability. The low stability of HepB3 may be surprising at the first sight, but it can only form two G-quartets; if we compare it with HepB1—which also has a two-quartet structure—HepB1 is more stable. We can hypothesize, that the last guanine of the HepB1 sequence can turn back and it can form a stability-increasing structure with two other guanines. This is not possible in case of HepB3, which results its lower stability.

### 3.2. Stabilizing Effect of Monovalent Cations

All the GQs have to be stabilized by metal ions that are located in the middle of the G-quartet, or in between of the quartets, depending on the geometrical constraints. We investigated four ions of the first column of the periodic table, namely: Li^+^, Na^+^, K^+^, Rb^+^. They have different ionic radii, which would predict differences in their stabilizing ability. We measured the temperature stability (i. e. the transition temperature, T_m_) for all the HepB oligos in the presence of all four ions. The results can be seen in Table 2.

As it can be seen, there is a difference between the values of HepB2 and the other GQs. This can be explained by the different sequences, which result in different structures. HepB2 contains four GGG repeats, while in the other two sequences there are GG sequences as well. This means, that HepB1 and HepB3 can only form a two-quartet GQ, while in HepB2 a three-quartet structure can be formed. Moreover, different cations can stabilize different conformations. In our earlier study we have shown, that HepB1 forms parallel structure in the presence of Li^+^, Na^+^ and Rb^+^, while K^+^ induces a hybrid structure. There is no common rule for the order of stabilization among the monovalent cations. Earlier studies suggested that K^+^ is the most potent stabilizer, followed by Na^+^ and Rb^+^ ions [51,52,53]. Li^+^ was reported to be too small to considerably stabilize the GQ form. Our results show somehow different order in stabilization. This can be explained by the fact, that earlier studies mainly focused on three-quartet GQ-forming, while HepB1 and HepB3—where the order of the stabilizing ions is different from the conventional one—can only form two-stage GQs. In case of two-stage GQs lithium is the most potent stabilizer. This implies a more closely packed structure, which can be stabilized by a relatively small ion, too. However, the differences in the stabilizing abilities of the ions are quite small. This fact suggests that the main factor in the stability of the quadruplexes is not the size of the central ion, but the hydrogen bonds stabilizing the GQ structure.

### 3.3. Pressure Experiments

#### 3.3.1. FRET Experiments under Pressure

Volumetric characterization of the GQs can be obtained from the pressure dependence of the system. The experiments described hereafter were performed in the presence of potassium ions, since this is the one that is present in highest concentration in vivo. The temperature unfolding experiments described above were also repeated at different elevated pressures. Unfortunately, this work is not as straightforward as it could be, using the pure thermodynamic description. This is due to the pH-dependence of the GQs. Although they are quite pH-insensitive, the slight pH-induced shift in the unfolding temperature can also lead to the distortion of the analysis.

This result inspired us to control and take into account the pH-alterations more carefully than is usually done. All of the experiments were pH-corrected, which means, that the pH was calculated for the actual pressure and temperature of the transition point. We used the dpH/dT values from Good et al. [54] This allowed us to perform a three dimensional fit afterwards, using the following function:

*T*_m_ = *a* + *b*⋅pH + *c*⋅*p*. (4)

**Figure 3:**
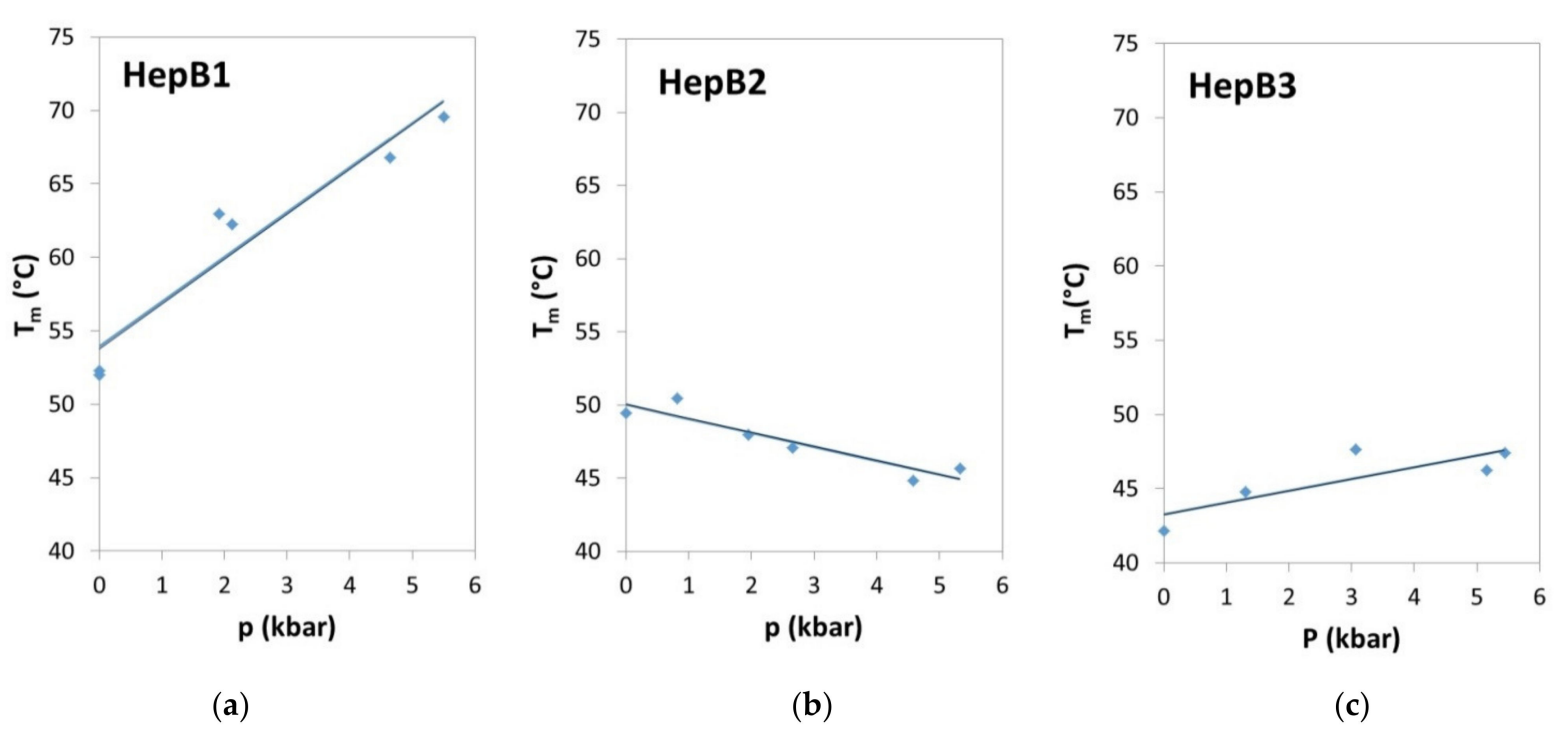
Pressure dependence of the unfolding temperature values for the three HepB variants. (**a**) HepB1, (**b**) HepB2, (**c**) HEpB3. The temperature values are pH-corrected as described in the text.

This way we obtained the pH- and pressure dependence of the unfolding temperature. Since the pH-dependence is not important, from the point of view of our volumetric study, and the three-dimensional plots are difficult to interpret, we made a pH-correction. (See the three-dimensional fits in Figures S2–S4) We corrected all the measurement points to the physiological pH value, pH = 7.4. Plots of the corrected T_m_ values as function of the pressure are shown in Figure 3. A linear fit was applied to the points to obtain the dT_m_/dp value, which was used to determine the volume change values. The slope of the line is actually the same as the *c* value deriving from the 3D fit (Equation (4)).

The volume change of the unfolding can be obtained from the slope of these lines using Equation (2). This also requires the Δ*H* values, which were obtained from the fit of the donor emission intensity data to Equation (1). The entropy changes decreased slightly with the pressure, therefore the value extrapolated to zero pressure was used. The Δ*H* values are: 192 ± 4 kJ/mol for HepB1, 124 ± 10 kJ/mol for HepB2 and 82 ± 9 kJ/mol for HepB3. The smaller enthalpy changes are reflected in the transition curves too; they are much less sharp, in the cases of HepB2 and HepB3, compared with HepB1.

Using these values, we can calculate the volume changes for each oligo.

#### 3.3.2. Unfolding of GQ as Seen from the Infrared Spectroscopy

FTIR spectra were recorded in the diamond anvil cell (DAC). The transmission cell has two diamond windows and requires a very small sample volume (few × 10 nl). However, the concentration of the sample should be much higher compared with the fluorescence experiments, in order to obtain good quality spectra. This is because the absorption of water in the infrared range limits the path length.

**Figure 4:**
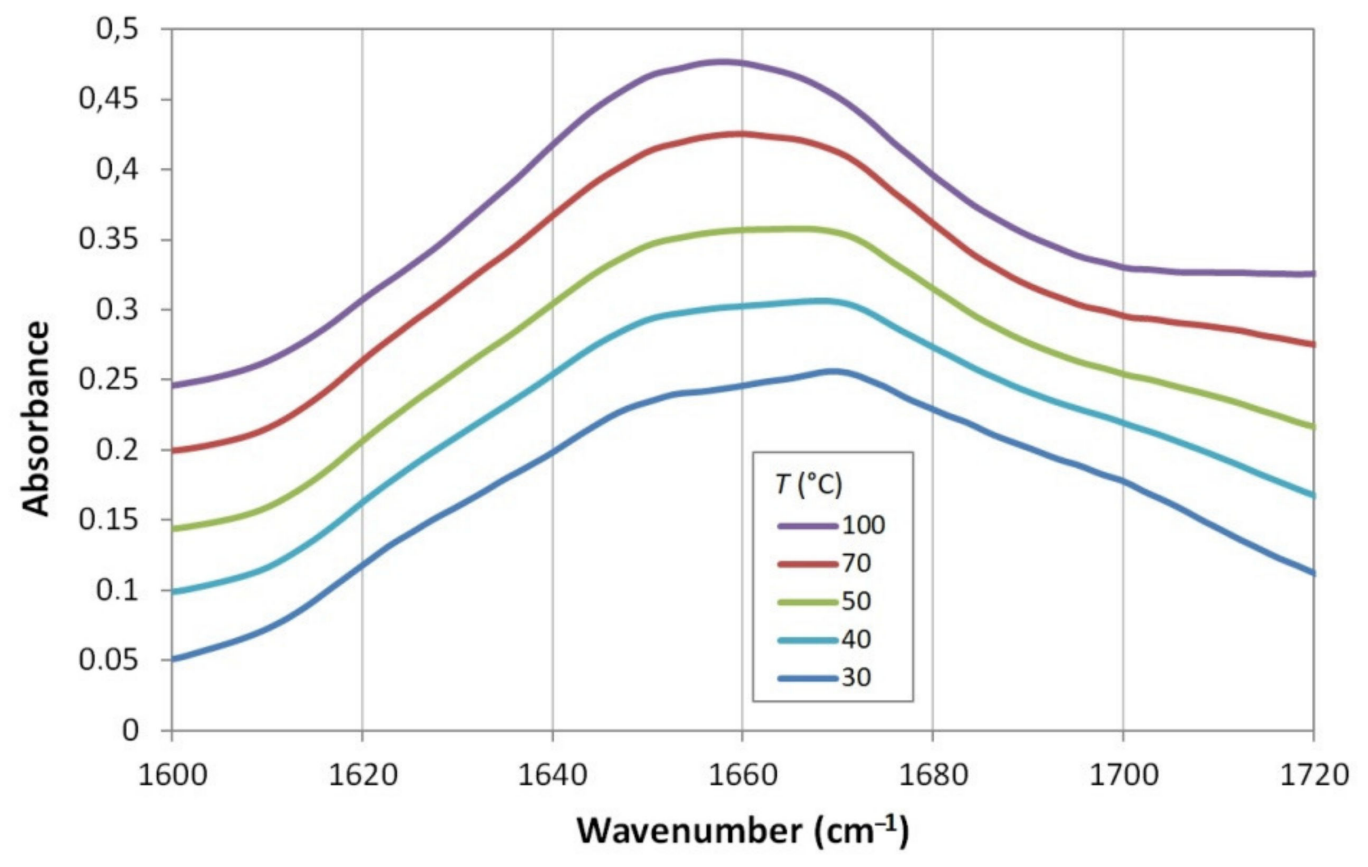
Infrared spectrum of HepB1 in the range of the C=O vibration at selected temperatures.

**Figure 5:**
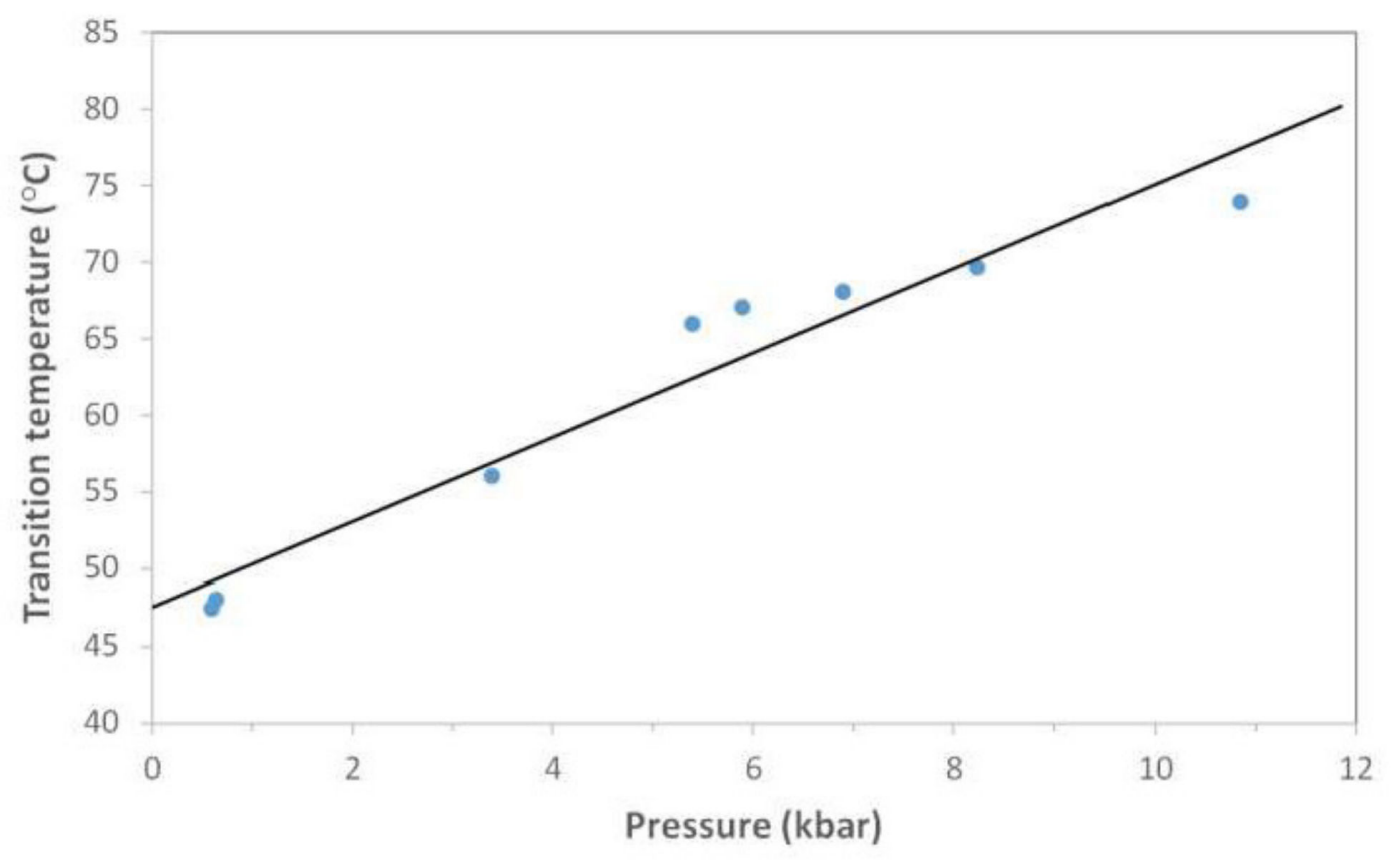
Transition temperatures of HepB1 versus pressure determined by FTIR spectroscopy.

The infrared spectrum contains absorption bands belonging to the molecular vibrations. The most conformation-sensitive vibration in the HepB GQs is the one in the range of 1660–1680. This band comes mainly from the C_6_ = O_6_ carbonyl vibration of the guanine base [42,55]. Since this takes part in the Hoogsteen-type hydrogen bond network, unfolding of the GQ alters the vibrational frequency [56]. Figure 4 shows this spectral region in case of HepB1 at a few selected temperatures. The position of the band is at 1660–1665 cm^−1^ for free guanine, it is near 1672 cm^−1^ in case of Hoogsteen-type hydrogen bonding, and it is at 1689 cm^−1^ in the Watson–Crick type double helix [42,57]. The presence of the 1672 cm^−1^ band in the low-temperature spectra is clear, and its disappearance in high-temperature spectra is also unequivocal. Simultaneously, the absorption in the middle of the 1660 cm^−1^ band is increasing. To determine the transition temperature, we fitted the absorbance values at 1660 cm^−1^ with Equation (1). We performed the same experiments at different pressure values. The transition temperatures are plotted against pressure in Figure 5. The Δ*V* value was obtained using calculations similar to the ones used for the fluorescence experiments earlier (see Table 3).

The other two HepB variants seemed to have different structure at high concentration needed for the infrared measurements. Their stability was considerably higher even at atmospheric pressure. This could be the sign of the stacking aggregation [58] or formation of the intermolecular GQs, which can be formed by two or four oligos. Therefore, we do not report their pressure behavior, here. The volume-change values obtained from the fluorescence and infrared experiments are in agreement in case of HepB1. One can, however, observe a slight curvature in both the fluorescence and infrared phase boundaries. This curvature points toward the possibility of the elliptic boundary, which is typical in case of proteins [16,19,59]. A similar phase diagram has been suggested by Chalikian and Macgregor [22] on the basis of theoretical calculations, but this is the first example, where such curved phase boundary is measured experimentally. Unfortunately, the experimental points cover only a small fraction of the ellipse; therefore, the fitting of the elliptic phase boundary is not possible. This is why we used the simple linear fit. Most probably, the rest of the ellipse lies out of the relevant and experimentally reachable region of the p-T diagram, if we take into account the freezing curve of the water [16,60].

#### 3.3.3. Volume Changes at the Unfolding of HepB GQs

**Scheme 1:**
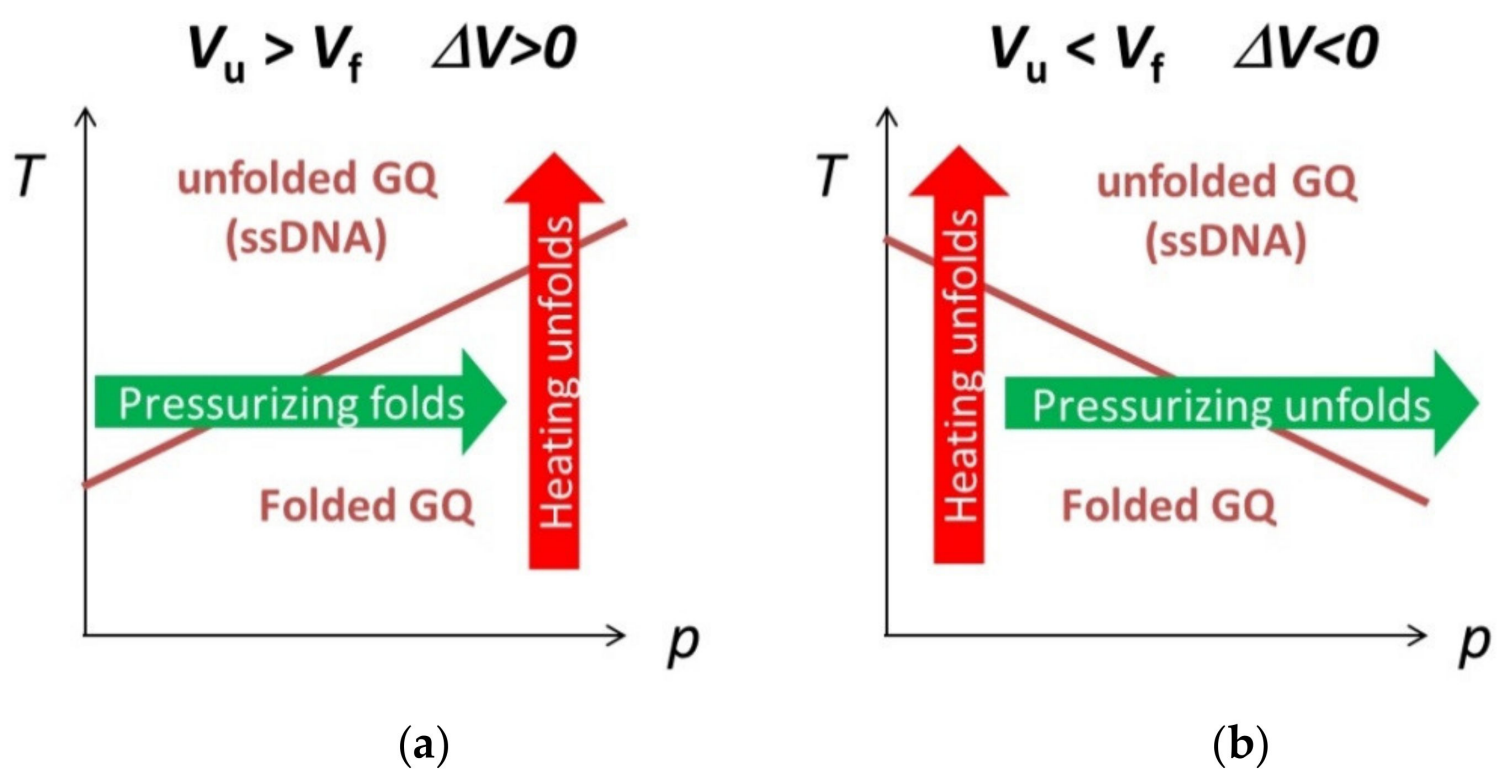
Shape of the temperature–pressure phase diagram, in the cases of different signs of the unfolding volume ∆*V*. (**a**) Δ*V* > 0 and (**b**) Δ*V* < 0. V_f_ and V_u_ are the volumes of the folded and unfolded molecules respectively.

As was mentioned at the end of Materials and Methods, Δ*V* is defined as the unfolding volume change. This is important to clarify, because not all the publications follow this convention. Positive Δ*V* means that the unfolded state has a larger volume compared with the folded one. This means that pressure favors the folded state. If the sample is pressurized, the unfolding temperature raises, i.e., d*T*_m_/d*p* > 0. The phase diagram looks like the one in Scheme 1a. If the phase boundary is crossed in the direction of the unfolding, a positive experimental Δ*V* can be obtained. If Δ*V* is negative, i.e., the folded state is more compact than the unfolded one; the phase-boundary line has a negative slope, d*T*_m_/d*p* < 0 (Scheme 1b). All these considerations are valid only if the unfolding is an endothermic reaction, as it is in the case of GQs [61].

A further important remark is that we always measure the volume change of our sample during the unfolding. Δ*V* is not only the volume change of the GQ itself, volume changes in the hydration shell contribute to it, as well.

As was mentioned earlier, the double-helix DNA does not really respond to pressure, due to its almost-zero unwinding volume. However, in GQs clear pressure effect has been measured for several different forms [25,42,49,62]. The rehydration of the coordinated cations and the volume changes of the DNA molecule itself are believed to be responsible for the volume change during the unfolding [49]. Measurements performed earlier using the thrombin binding aptamer (TBA) showed negative unfolding volume change [41]: Δ*V* = −55 ± 4 cm^3^/mol. For Htel, −92 ± 5 cm^3^/mol was measured by Takahashi et al. [62]. These authors observed reduction of the Δ*V* in the presence of crowding agents such as polyethylene glycol. Considerably smaller value (−43 ± 7 cm^3^/mol) was measured by Li et al. [49]. In our earlier experiments, we investigated four human GQs—c-MYC, KIT, VEGF and Htel—wherein we obtained Δ*V* = −17 ± 1, −6 ± 1, −18 ± 5, −19 ± 3 cm^3^/mol values respectively [25,42]. The variation in the experimental values points to the importance of slight environmental parameters like crowding, concentration, etc. We have also noticed (looking into the details of the presumably multistep process) that different experimental techniques can provide slightly different transition temperatures.

As mentioned earlier, the rehydration of the central ions was suggested to be responsible for the pressure sensitivity of GQs. Rehydration decreases the volume of the system, since the hydration shell is denser than the bulk water. It could not be, however, the only contribution, because similar values were measured for the GQs containing the same number of quartets, consequently the same number of stabilizing ion. Chalikian’s group studied the volumetric aspects of the GQ folding in the case of Htel [63]. They distinguished four terms in the volume change:

Δ*V* = Δ*V*_M_ + Δ*V* _T_ + Δ*V*_H_ + n*V*_K+_ (5)

where Δ*V* is the measured unfolding volume change, Δ*V*_M_ is the molecular volume, i.e., the volume impenetrable to other molecules, Δ*V*_T_ is the thermal volume, which is a result of molecular vibrations and imperfect packing, Δ*V*_H_ is the volume change due to the uptake of water molecules into the hydration shell, and *V*_K+_ is the partial molar volume of the K^+^ ion. (Our equation differs from the Equation (4) of ref [63] since they study the folding, while we describe the unfolding volume changes.)

Their calculations based on Htel experiments show more than ten-times higher hydration volume change (779 cm^3^/mol), compared with the experimental value (69 cm^3^/mol). This was compensated however by high thermal volume of the opposite sign. Δ*V*_M_ and *V*_K+_ are small (6 and 6.3 cm^3^/mol, respectively) compared with the other two terms. This means that the volume change of GQs is a delicate balance between hydration and thermal volumes.

According to the results of our pressure experiments we obtained both negative and positive volume changes. As it was mentioned earlier, HepB1 and HepB3 can form two-quartet GQs, while HepB2 is able to form three G-quartets. This correlates with the signs of the volume change values, which is negative only in case of the three-quartet GQ. The positive unfolding volumes in case of HepB1 and HepB3 are unusual for the GQs. There is however a unique feature in these sequences: they contain longer G repeats with three and four guanines. Since there are only two quartets, not all of the guanines of such long G repeats can participate in the stabilization of the GQ form; they might even destabilize the GQ structure and can be responsible for the positive volume changes.

### 3.4. Stabilization of the HepB GQs with TMPyP4

The cationic porphyrin compound TMPyP4 was developed to stabilize the human telomere GQ, in order to inhibit the telomerase enzyme, which elongates the telomere region of the DNA, contributing to the immortality of cancer cells. We investigated whether this porphyrin molecule is able to stabilize the GQs in the genome of the hepatitis B virus. The porphyrin was added to the FRET-labeled GQs at a four-fold excess. The unfolding temperature increased considerably for all the HepB variants. In some cases, the transition shifted out of our experimental range, which only allowed us to obtain a lower estimate for the stabilization. Table 4 shows the extent of stabilization.

As can be seen, all the HepB GQs are considerably stabilized by the binding of TMPyP4. One can, however, ask as to whether the ligand binds to the unlabeled oligos, too, or only to the FRET-labeled ones. The binding of TMPyP4 to the unlabeled HepB GQs can be proven by a competitive binding assay, similar to the one described by Luo [64]. The principle is, briefly: 1. The unfolding temperature of a FRET-labeled oligo is measured (T_m1_). 2. The increased unfolding temperature (T_m2_), in presence of the ligand, is determined. 3. Finally, the sample containing both the labeled and unlabeled oligo, together with the ligand, is measured. If the ligand binds to the unlabeled oligo, the fluorescence curve in the third and first experiments are similar, the T_m3_ is near to T_m1_ rather than to T_m2_. The results are shown in Figures S5–S7. Our results show clearly that TMPyP4 binds to all tree unlabeled oligos, however, with different strength. HepB2 binds TMPyP4 with smaller affinity than the others, as can be seen from the results of the competitive study.

The stabilization of HepB oligos by TMPyP4 points toward practical applications: TMPyP4 can shift the equilibrium between the double helix and the GQ form and might hinder the transcription and the proliferation of the virus.

## 4. Conclusions

We found that all of the three potential GQ-forming sequences of the hepatitis B virus fold into a GQ structure. They can be stabilized by Li^+^, Na^+^, K^+^, and Rb^+^. Lithium is the most potent stabilizer for the two-quartet GQs, formed by the HepB1 and HepB3 oligos. This suggests that these GQs have a more closely packed structure that allows the stabilization by the small Li^+^ ion. Pressure studies resulted in a positive unfolding volume for HepB1, while, for HepB2 and HepB3, we obtained small negative and positive values. Volumetric parameters are important factors in crowded environment, which is typical for biological systems. Under this molecular crowding condition, the available volume is limited, which can shift the folding equilibrium of GQs. All the HepB oligos were stabilized by TMPyP4, which was developed to increase the stability of the human telomere GQ. The stabilization effect was very pronounced—more than 20 °C—which points toward practical applications: TMPyP4 might shift the equilibrium between the double helix and the GQ form and might hinder the transcription of the virus. This might have a medical importance, since the stabilization of GQs in the viral DNA shifts the equilibrium between the double helix and the GQ form, which is believed to influence the transcription of the genome.

## Figures and Tables

**Table 1:** Relative fluorescence efficiency values of HepB oligos in folded and unfolded conformations. All experiments were performed in presence of potassium ion.

Oligo Name	Relative FRET Efficiency *E*_rel_	
	Folded	Unfolded
HepB1	0.65	0.21
HepB2	0.64	0.27
HepB3	0.69	0.24

**Table 2:** Transition temperature (T_m_) of the three studied HepB oligos in presence of different cations. The order of stabilizing ability is indicated as well.

Oligo	Li^+^	Na^+^	K^+^	Rb^+^	Stabilizing Order
HepB1	58.5	56.6	53.8	54.4	Li^+^ > Na^+^ > Rb^+^ ≳ K^+^
HepB2	46.9	51.0	51.9	45.6	K^+^ > Na^+^ > Li^+^ ≳ Rb^+^
HepB3	49.4	48.1	41.4	44.4	Li^+^ > Na^+^ > Rb^+^ > K^+^

≳ means a little bit larger.

**Table 3:** Pressure dependence of unfolding temperature and the calculated volume-change values for the HepB oligos in the presence of potassium ions.

Oligo Name	Technique	dT_m_/dp (°C/kbar)	dV (cm^3^/mol)
HepB1	FRET	3.04 ± 0.58	17.8 ± 3.8
	IR	2.75 ± 0.28	16.1 ± 2.0
HepB2	FRET	−0.96 ± 0.22	−3.7 ± 0.6
HepB3	FRET	0.79 ± 0.38	2.0 ± 1.1

**Table 4:** Temperature stabilization of HepB variants by the cationic porphyrin TMPyP4. All experiments were performed in the presence of potassium ion.

	Oligo Name		
	HepB1	HepB2	HepB3
Δ*T*_m_ (°C) = T_m,HepB+TMPyP4_ − T_m,HepB_	23	>36	>47

## Data Availability

The data presented in this study are available on request from the corresponding authors.

## Supplementary Materials

The following are available online at https://www.mdpi.com/article/10.3390/biology10111173/s1, Figure S1: CD spectra of HepB2 (a) and HepB3 (b) in presence of K^+^ ion. Figures S2–S4: Three dimensional fits of unfolding parameters of HepB1, HepB2 and HepB3 in the pressure-temperature-pH parameter space. Figures S5–S7: Competitive binding assays of HepB1, HepB2 and HepB3.
