# Supplementary material for: Pressure Perturbation Studies of Noncanonical Viral Nucleic Acid Structures
Source: Biology (Basel). 2021 Nov 12;10(11):1173. doi: 10.3390/biology10111173 (PMC8615049; doi:10.3390/biology10111173)
Supplement: Supplementary file 1 [file biology-10-01173-s001.zip › biology-1422139-supplementary.pdf]

Supplementary Information for:

Biology **2021**, *10*, 1173. https://doi.org/10.3390/biology10111173

Pressure perturbation studies of noncanonical viral nucleic acid structures

Judit Somkuti, Orsolya Réka Molnár, Anna Grád and László Smeller*

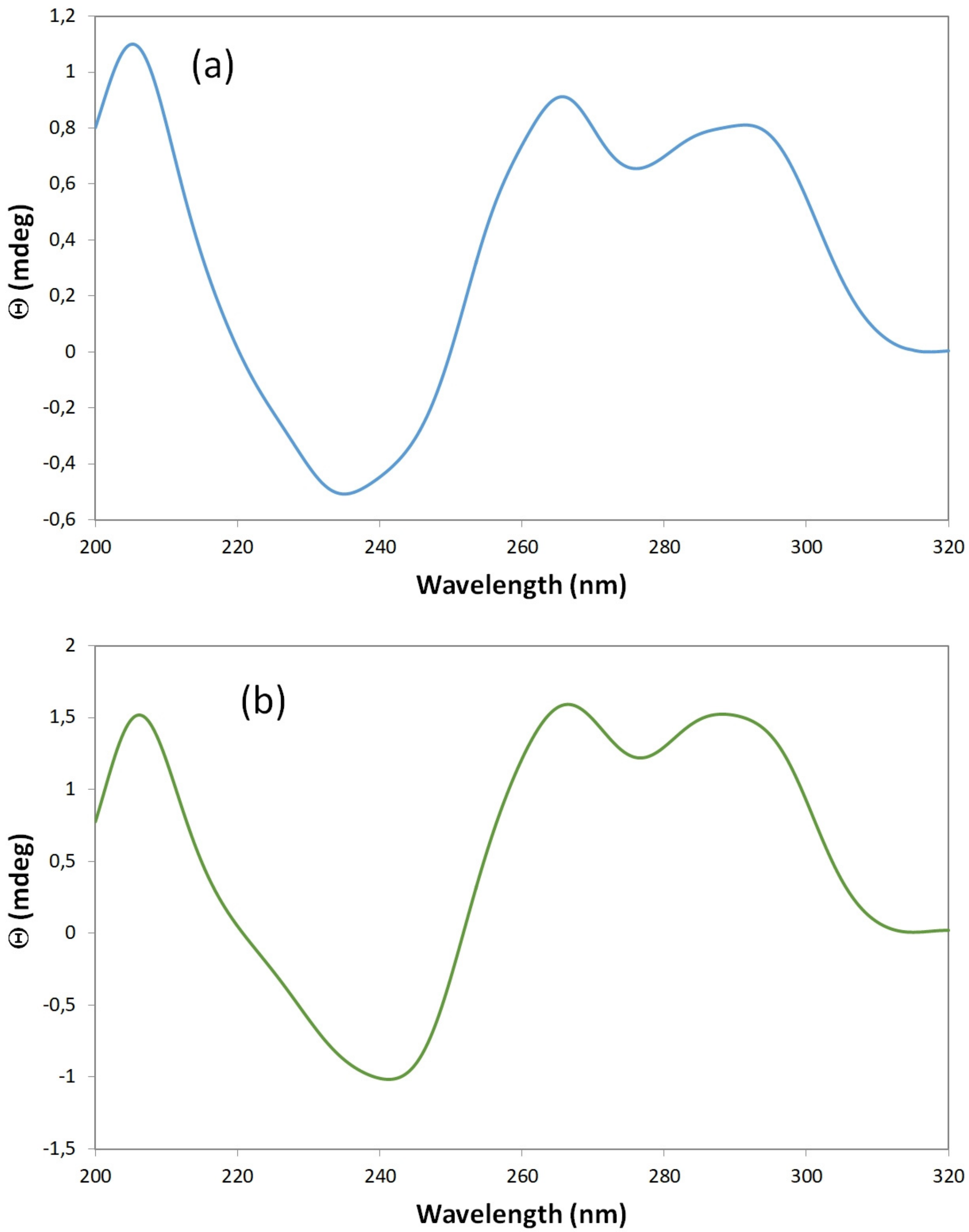

Figure S1. CD spectra of HepB2 (a) and HepB3 (b) in presence of K^+ ion.

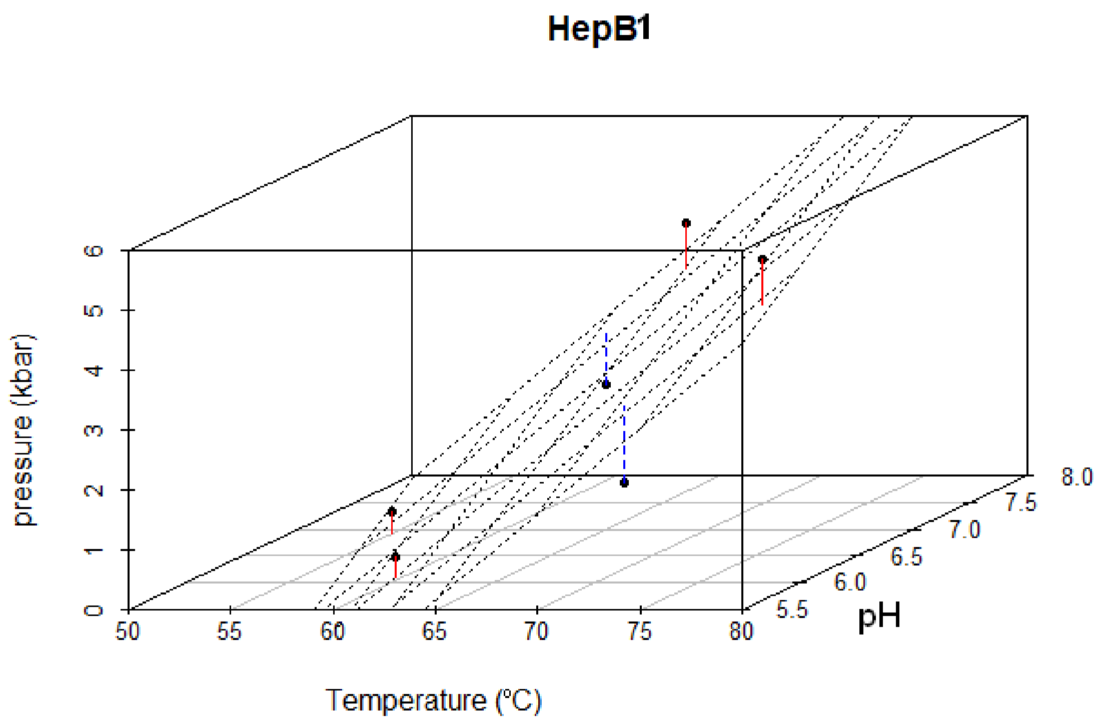

Figure S2. Three dimensional fit of unfolding parameters of HepB1 on the pressure-temperature-pH parameter space. Dots represent the experimental results, their deviation from the fitted plane are indicated by red and blue lines according to the sign of the deviation. The fitted plane is shown by dotted gridlines.

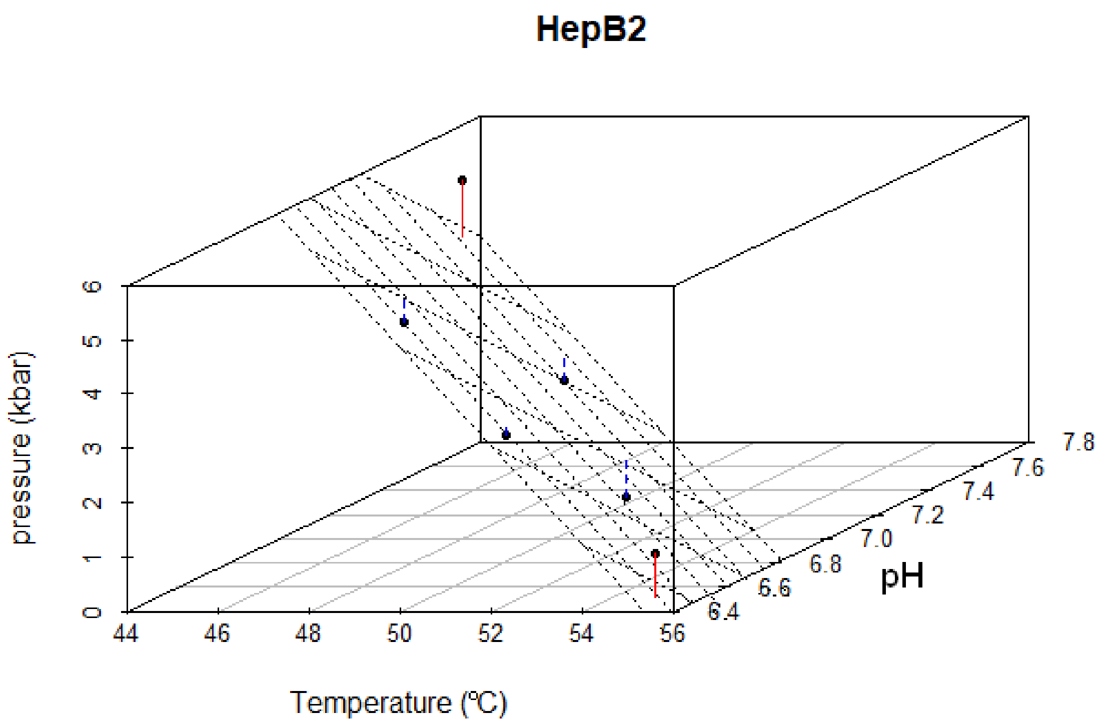

Figure S3. Three dimensional fit of unfolding parameters of HepB2 on the pressure-temperature-pH parameter space. Dots represent the experimental results, their deviation from the fitted plane are indicated by red and blue lines according to the sign of the deviation. The fitted plane is shown by dotted gridlines.

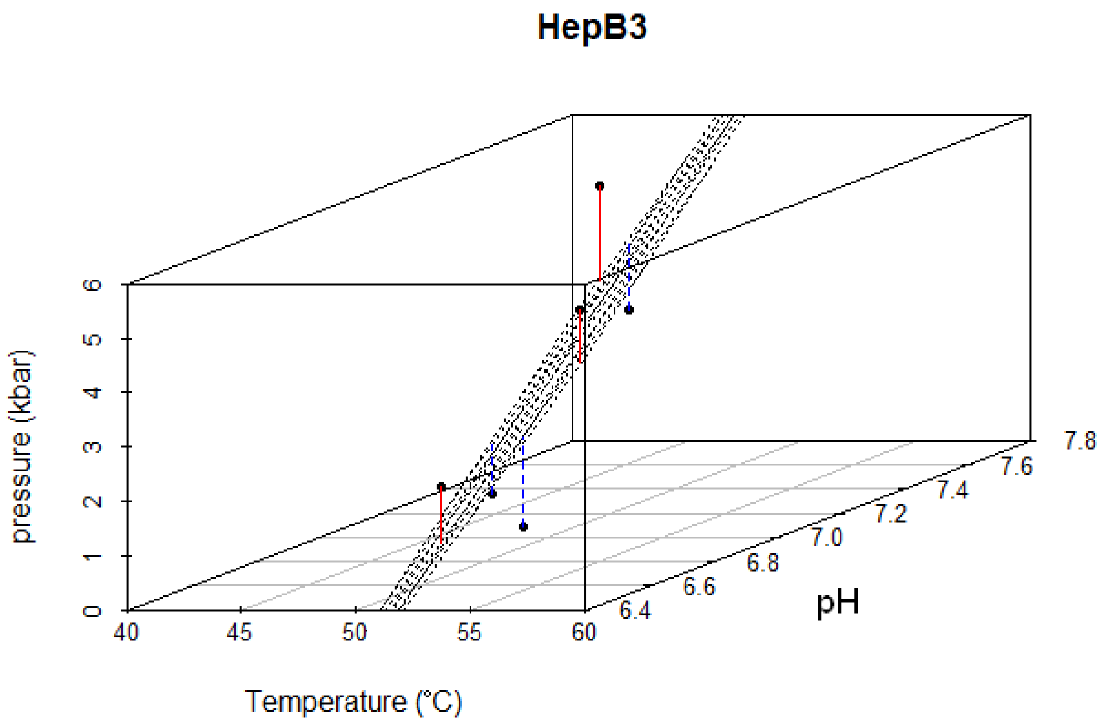

Figure S4. Three dimensional fit of unfolding parameters of HepB3 on the pressure-temperature-pH parameter space. Dots represent the experimental results, their deviation from the fitted plane are indicated by red and blue lines according to the sign of the deviation. The fitted plane is shown by dotted gridlines.

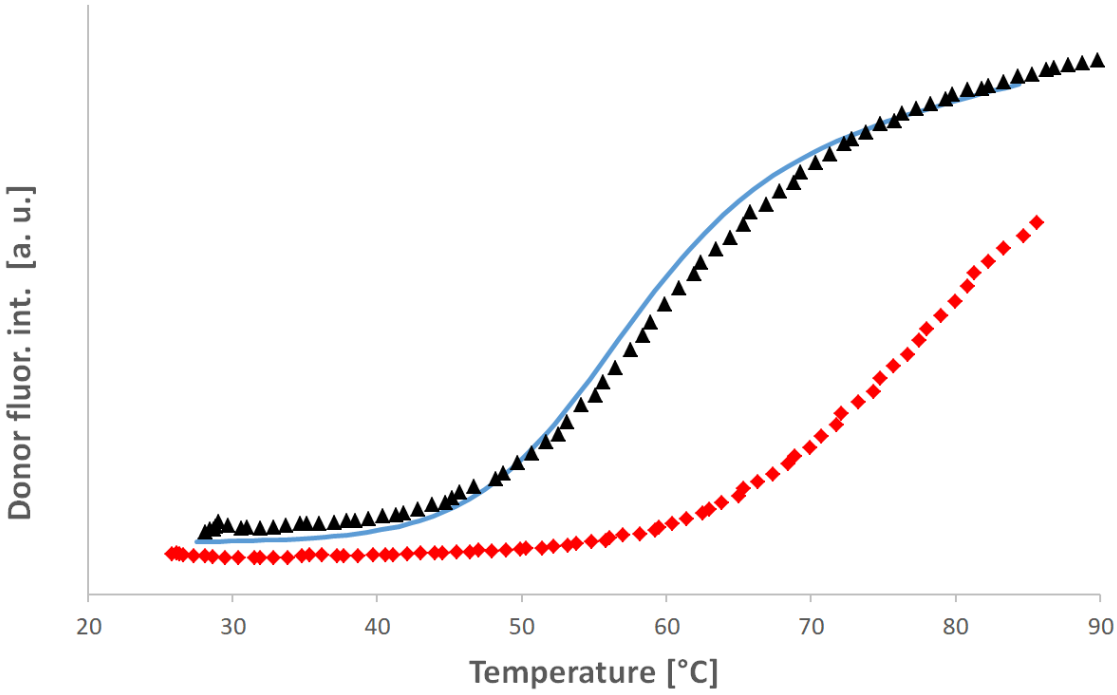

Figure S5. Comptetitive binding assay of HepB1. Blue line shows the transition curve of the HepB1_FRET oligo itself (the same as in inset of fig. 2a). Red diamonds show the case of HepB1_FRET+TMPyP4, whole the black triangles show the HepB1_FRET+HepB1(unlabeled)+TMPyP4.

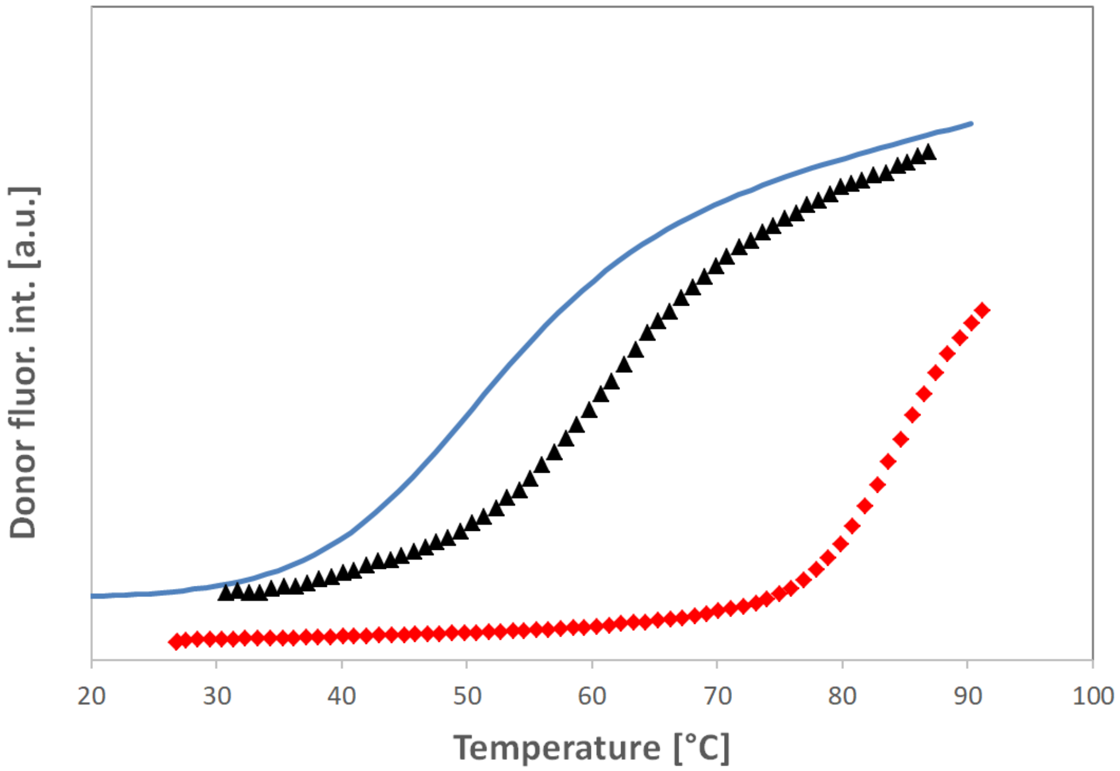

Figure S6. Comptetitive binding assay of HepB2. Blue line shows the transition curve of the HepB2_FRET oligo itself (the same as in inset of fig 2b). Red diamonds show the case of HepB2_FRET+TMPyP4, whole the black triangles show the HepB2_FRET+HepB2(unlabeled)+TMPyP4.

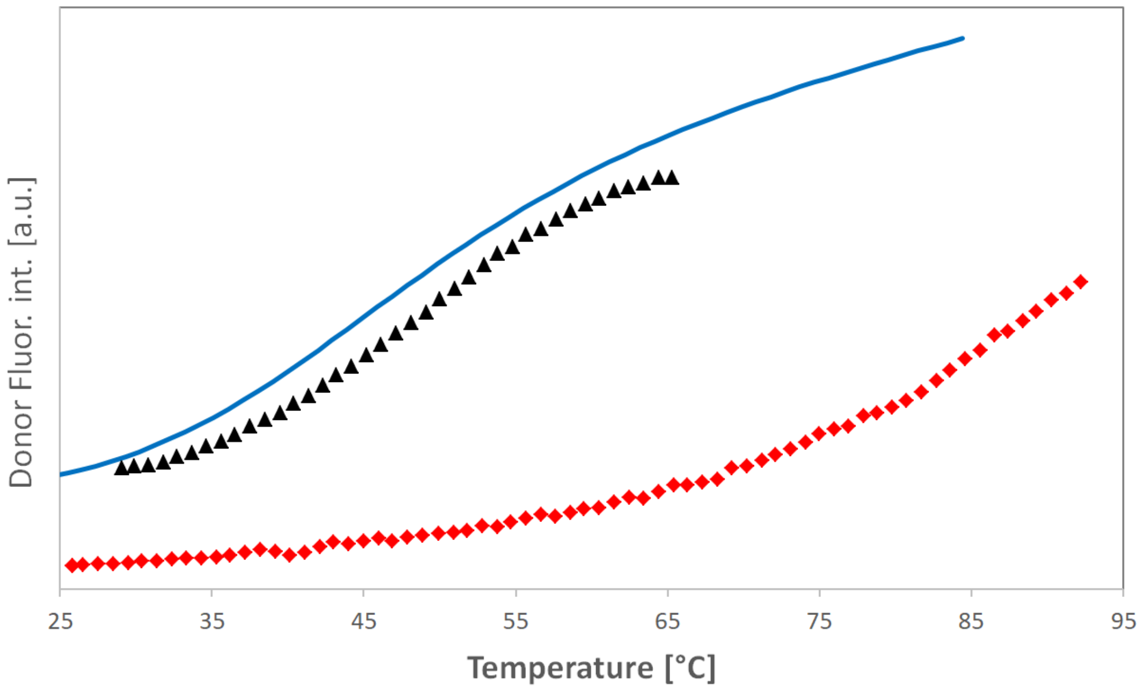

Figure S7. Comptetitive binding assay of HepB3. Blue line shows the transition curve of the HepB3_FRET oligo itself (the same as in inset of fig 2c). Red diamonds show the case of HepB3_FRET+TMPyP4, whole the black triangles show the HepB3_FRET+HepB3(unlabeled)+TMPyP4.
